# Design and Synthesis of Imidazopyrazolopyridines as Novel Selective COX-2 Inhibitors

**DOI:** 10.3390/molecules200815287

**Published:** 2015-08-21

**Authors:** Mohamed G. Badrey, Hassan M. Abdel-Aziz, Sobhi M. Gomha, Mohamed M. Abdalla, Abdelrahman S. Mayhoub

**Affiliations:** 1 Chemistry Department, Faculty of Science, Fayoum University, El-Fayoum 63551, Egypt; E-Mail: mgb00@fayoum.edu.eg; 2 Department of Chemistry, Faculty of Science, Bani Suef University, Bani Suef 62111, Egypt; E-Mail: dr_hassan1971@yahoo.com; 3 Department of Chemistry, Faculty of Science, Cairo University, Giza 12613, Egypt; 4 Research Unit, Saco Pharm. Co., 6th of October City 68330, Egypt; E-Mail: dr_mohamed211@yahoo.com; 5 Department of Organic Chemistry, Faculty of Pharmacy, Al-Azhar University, Cairo 11884, Egypt; E-Mail: amayhoub@hotmail.com

**Keywords:** aminopyrazolopyridine, anti-inflammatory, cyclooxygenase, hydrazonyl halides, selective inhibitors

## Abstract

The usefulness of non-steroidal anti-inflammatory drugs (NSAIDs) is hampered by their gastrointestinal side effects. Non-selective cyclooxygenases inhibitors interfere with both COX-1 and COX-2 isozymes. Since COX-1 mediates cytoprotection of gastric mucosa, its inhibition leads to the undesirable side effects. On the other hand, COX-2 is undetectable in normal tissues and selectively induced by inflammatory stimuli. Therefore, it is strongly believed that the therapeutic benefits derive from inhibition of COX-2 only. The presence of a strong connection between reported COX-2 inhibitors and cardiac toxicity encourages medicinal chemists to explore new scaffolds. In the present study, we introduced imidazopyrazolopyridines as new potent and selective COX-2 inhibitors that lack the standard pharmacophoric binding features to *h*ERG. Starting from our lead compound **5a**, structure-based drug-design was conducted and more potent analogues were obtained with high COX-2 selectivity and almost full edema protection, in carrageenan-induced edema assay, in case of compound **5e**. Increased bulkiness around imidazopyrazolopyridines by adding a substituted phenyl ring(s) afforded less active compounds.

## 1. Introduction

Non-steroidal anti-inflammatory drugs (NSAIDs) are commonly used for the treatment of inflammation, pain, and fever. From mechanistic point of view, NSAIDs exert their pharmacological action via inhibition of cyclooxygenase (COX) that catalyzes the conversion of arachidonic acid to the prostaglandins (PGs) [1]. PGs are hormone-like bio-substances that mediate different signaling pathways in many physiological and pathological processes. COX has been established to exist mainly in two distinct isoforms, COX-1 and COX-2. COX-1 is constitutively normally expressed in most tissues, and PGs controlled by COX-1 mediate cytoprotection of gastric mucosa and platelet aggregations in addition to some other physiological processes. On the other hand, COX-2 is undetectable in normal tissues and selectively induced locally by inflammatory stimuli; *i.e.*, pro-inflammatory cytokines, leading to elevating PG levels at the site of inflammation [2,3]. Therefore, the therapeutic benefits derives from inhibition of the inducible isoform; *i.e.*, COX-2, at the site of inflammation [1].

Overexpression of COX-2 is not limited by rheumatic inflammation but extends to mycobacterial pulmonary inflammation [4], vascular inflammation [5], cigarette-induced airway inflammation [6], and *Helicobacter pylori* induced gastritis [7]. It also plays a pivotal pathological role in intestinal inflammation and colorectal cancer [8]. Moreover, connection between COX-2 and different types of cancer is recently reported via different mechanisms [9,10,11,12,13,14].

Commonly used NSAIDs are non-selective in their action and inhibit both COX-1 and COX-2 explaining toxicity-mediated by inhibition of the non-regulated COX-1, in normal cells. However, NSAIDs are clinically effective in pain and inflammatory relief, their use is hampered by significant side-effects (mainly in GIT) due to inhibition of COX-1 [15]. In contrast to other NSAIDs, selective COX-2 inhibitors do not cause notable ulcers in the stomach or intestine, they are active as non-selective NSAIDs and inhibit PG synthesis in inflammatory cells [15,16,17]. So far, it was believed that “the more selective COX-2, the less side effects”.

Chemical structures of reported COX-2 inhibitors are highly diverse. In general, they are, unlike classical NSAIDs, lacking the free carboxylate and could be classified into: (1) Carbocycles and Heterocycles with Vicinal Aryl Moieties; (2) Diaryl or Aryl/Heteroaryl-Ether and -Thioether Derivatives; (3) *cis*-Stilbene Derivatives; (4) Diaryl and Aryl/Heteroaryl Ketones [18]. Regardless the relatively large number of approved COX-2 inhibitors, there is only one selective COX-2 inhibitor in the US market (Celecoxib) [19]. Recent market removal of some selective COX-2 inhibitors such as rofecoxib due to its cardiac toxicity [20] encourages medicinal chemists to explore other alternative scaffolds. The cardiotoxicity of the withdrawn COX-2 inhibitors was attributed mainly to their *h*ERG affinity [21]. In our attempt to find a different selective COX-2 scaffold, we chose pyrazolopyridine that recently showed great inhibitory selectivity towards COX-2 [22,23,24]. One advantage of such new ring system is lacking the putative pharmacophoric criteria of *h*ERG binders [25]. Virtual screening of our in-house library of fused pyrazolopyridines, pyrazolopyrimidines, imidazopyridines, and imidazopyrimidines, using Sybyl-X, furnished compound **5a** as a potential hit structure. Compound **5a** revealed an acceptable COX-2 inhibitory activity with 9.4 times selectivity towards the isozyme-2 (Table 1).

**Figure 1:**
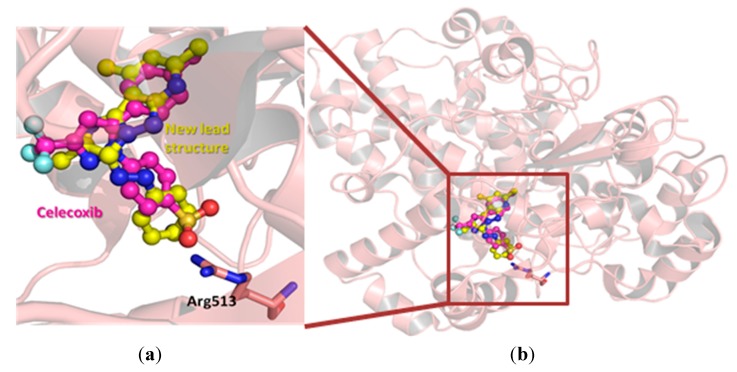
The hypothetical binding mode of the lead compound **5a** (colored yellow) within COX-2 active site (PDB ID:3ln1), co-crystalized with celecoxib (colored magenta). The important Arg 513 residue was highlighted as sticks in Figure 1a.

Research Design: To explain the moderate COX-2 inhibitory activity and selectivity of the lead structure **5a**, a modeling study was conducted using a co-crystal structure of a selective COX-2 inhibitor, celecoxib, within COX-2 active site (PDB ID:3ln1 [26]). Celecoxib was extracted and compound **5a** was docked within COX-2 active pocket using GOLD (Figure 1a). Interestingly, our newly defined lead structure **5a** showed great overlap with celecoxib binding conformation (Figure 1b); *i.e.*, the imidazopyrazolopyridine was found to overlay the phenylpyrazole structure of celecoxib, while the phenyldiazene moiety of **5a** extends parallel to benzenesulfonamide moiety of celecoxib as shown in Figure 1.

**Figure 2:**
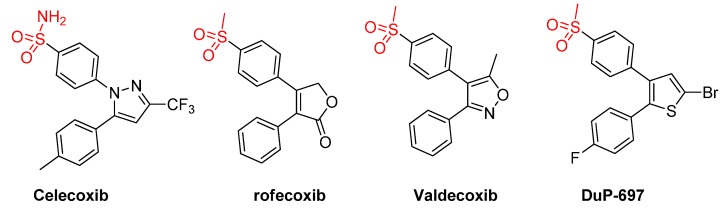
Chemical structures of some selective COX-2 inhibitors (coxibs).

One key difference between the two cyclooxygenases is the size of their active pockets. COX-2 has a larger binding pocket, where three bulky amino acid residues: Ile523, His513, and Ile434 were replaced with less-sized residues (Val523, Arg513, and Val434) [27]. Therefore, all early discovered COX-2 inhibitors contain diaryl rings with *cis*-stilbene-like configuration to reduce binding affinity towards the less-volume binding site of COX-1 [18,28]. The higher degree of aromatic structure superposition between our lead compound and those of celecoxib might explain its COX-2 selectivity (Figure 1a). To improve the inhibitory potency and selectivity, medicinal chemists usually add a hydrogen-bond acceptor moiety to one aromatic ring as shown in Figure 2. This hydrogen-bond acceptor targets Arg513, which is replaced by a histidine residue in the case of COX-1 [27]. Analogously, we decided to explore the effect of tethering a hydrogen-bond acceptor to the phenyl ring of our newly discovered lead structure **5a**. In addition, we also decided to increase the bulkiness degree around imidazopyrazolopyridine nucleus in order to improve compounds’ selectivity.

## 2. Results and Discussion

### 2.1. Chemistry

Generally, pyrazoplopyridines were reported to be prepared through two alternative routes, either from building up pyrazole moiety first, and then allowed it to react with activated nitriles [29]; the second route involved constructing polyfunctional pyridine nucleus, followed by reaction with various electrophilic reagents [30]. In the present work, we utilized aminopyrazolopyridine **1** as a key precursor for synthesis of our library of COX-2 inhibitors. Aminopyrazolopyridines **1** were chosen as starting building blocks because of their synthetic accessibility and affordable commercial availability of their starting materials.

**Scheme 1:**
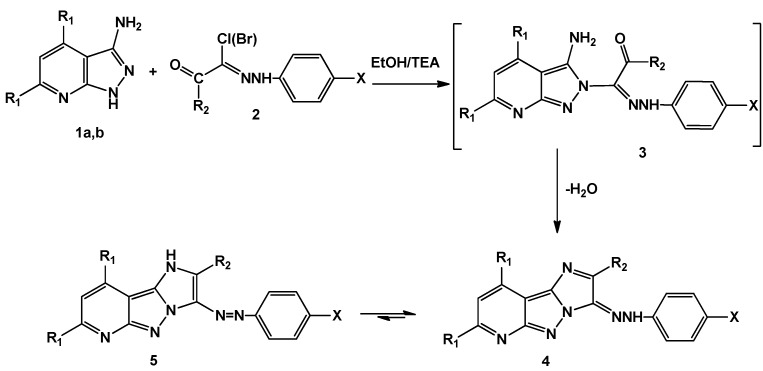
Synthesis of 2,7,9-trisubstituted-3-(aryldiazenyl)-3*H*-imidazo[1′,2′:1,5] pyrazolo[3,4-*b*]pyridine **(5a**–**l)**.

When aminopyrazolopyridines **1** were allowed to react with a variety of hydrazonyl halides **2** in basic medium, a removal of hydrogen halide followed by cyclization through water loss led, finally, to the imidazopyrazolopyridines **5a**–**l** (Scheme 1).

**Table:** 

Compd. No.	R_1_	R_2_	X	Compd. No.	R_1_	R_2_	X
**a**	Me	Me	H	**g**	Ph	Me	OMe
**b**	Me	Me	Cl	**h**	Ph	Me	Cl
**c**	Me	Me	OMe	**i**	Ph	Me	Br
**d**	Me	Me	Br	**j**	Ph	Me	NO_2_
**e**	Me	Me	NO_2_	**k**	Ph	Ph	H
**f**	Ph	Me	H	**l**	Ph	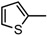	H

Elucidation of all structures was assessed from their elemental analyses and spectral data. In general, the newly built imidazole ring was confirmed by disappearance of the NH_2_ signal characteristic to the starting material from ^1^H-NMR spectrum, as well as loss of the corresponding bands in the IR spectrum. In addition, no absorption bands in carbonyl group characteristic region were observed, which in turn confirmed the cyclization step. The products exist in two tautomeric forms (azo-hydrazo forms) with equilibrium shifted mainly towards the azo form as indicated by the ^1^H-NMR spectra. For example the ^1^H-NMR spectrum of **5a** displayed a characteristic D_2_O-exchangeable singlet signal at 11.16 ppm attributable to imidazole NH proton (azo-structure). The ^1^H-NMR spectrum of compound **5g** displayed two singlet signals at 2.26 and 3.81 for two methyl groups. In addition, the aromatic region showed aggregated peaks from 7.13–8.27 ppm integrating for 15 protons (one pyridine proton plus phenyl protons). The mass spectrum of this derivative gave the corresponding molecular ion peak at *m*/*e* = 458. Furthermore, when R_1_ = R_2_ = Ph and X = H (compound **5k**), the ^1^H-NMR spectrum showed a nice singlet at δ = 6.95, which might be attributable to pyridine H at position-3. The aromatic region is integrating for 20 protons spread over a smaller chemical shift range from 7.01 to 7.38 ppm, because all aromatic protons are almost chemically equivalent (phenyl ones).

**Scheme 2:**
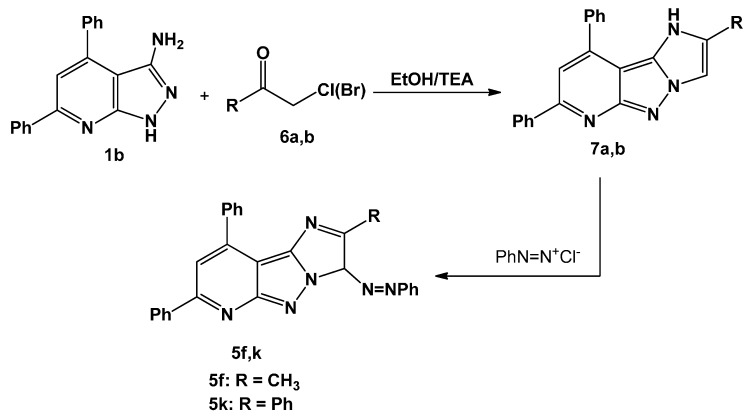
Synthesis of imidazo[1′,2′:1,5]pyrazolo[3,4-*b*]pyridine (**5f** and **5k**).

Alkylation of compound **1b** with α-haloketones such as chloroacetone and phenacyl bromide in basic medium resulted in heterocyclization and afforded the tricycles compounds **7a**,**b**. As compounds **7a**,**b** were coupled to benzene diazonium chloride, they delivered the desired products **5f**,**k** (Scheme 2); this could be considered as a chemical evidence for the structures of the products obtained from reaction of **1b** with hydrazonyl halides. The mass spectra of compounds **7a**,**b** gave the correct molecular ion peaks for the proposed structures, in addition, ^1^H-NMR spectrum of compound **7a** showed a singlet at 2.37 ppm (CH_3_ protons) and a singlet signal at 11.71 ppm (NH).

### 2.2. Biological Discussion

Cyclooxygenase Inhibition Activity: all synthesized compounds were subjected to colorimetric screening assay utilizing the peroxidase component of cyclooxygenases (COX-1 and COX-2). The peroxidase activity is assayed colorimetrically as detailed in the experimental section. The results were summarized in Table 1.

**Table 1:** Cyclooxgenanse (COX-1 and COX-2) inhibitory activity.

Compd. No.	Drug in μg/mL	Percentage Inhibition *		COX-2/COX-1 Selectivity
		COX-1	COX-2	
**5a**	10	3.88 ± 0.0023	36.51 ± 0.35	9.4
**5b**	10	3.84 ± 0.0034	59.11 ± 0.44	15.4
**5c**	10	3.76 ± 0.0023	68.34 ± 0.53	18.2
**5d**	10	3.80 ± 0.0032	67.72 ± 0.44	17.8
**5e**	10	3.92 ± 0.0023	75.92 ± 0.36	19.4
**5f**	10	3.96 ± 0.0034	25.31 ± 0.40	6.4
**5g**	10	3.99 ± 0.0025	24.72 ± 0.59	6.2
**5h**	10	3.62 ± 0.0036	20.85 ± 0.38	5.6
**5i**	10	3.58 ± 0.0025	11.50 ± 0.47	3.2
**5j**	10	3.54 ± 0.0034	22.15 ± 0.65	6.6
**5k**	10	3.51 ± 0.0046	12.80 ± 0.74	3.6
**5l**	10	3.69 ± 0.0027	9.58 ± 0.83	2.5
**Diclofenac**	10	56.78	7.88	0.13
**Valdecoxib**	10	12.15	76.15	6.26
**Ibuprofen**	10	78.89	5.23	0.06

Values were calculated from the mean values of data from three separate experiments and presented as mean ± S.E.M.; * The percentage Inhibition is the percent in inhibition of the either COX enzyme activities at the selected doses; All results are significant different from control values at *p* ≤ 0.005; All results are significant different from reference standard values at *p* ≤ 0.005.

**Figure 3:**
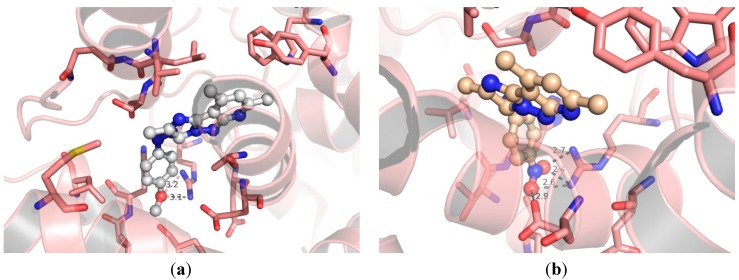
Hypothetical binding modes of compound **5c** (**a**); and **5e** (**b**) in COX-2 protein (PDB ID:3ln1). Dashed lines represent distances between heavy atoms.

The lead compound **5a** showed moderate COX-2 inhibitory activity and low affinity towards COX-1 (Table 1). Adding a hydrogen-bond acceptor para to the phenyl ring connected with the diaza moiety gave compounds **5b**–**e**. The key idea behind inserting hydrogen-bond acceptors at this particular position is targeting Arg513, which is replaced by a histidine residue in the case of COX-1 [27], as detailed earlier. Again, targeting Arg513 was believed to ameliorate COX-2 selectivity. A Chlorine-containing derivative **5b** showed enhanced COX-2 inhibitory activity; *i.e.*, the percentile value of COX-2 inhibition jumped up from 36.5 in the case of **5a** to 59.1 in the case of **5b**, and selectivity towards COX-2 over COX-1 is also increased to 15 times (Table 1). Replacement chlorine atom with methoxy group afforded compound **5c** that revealed better selectivity toward COX-2; *i.e.*, **5c** is 18 times more selective. Compound **5c** was on par with the lead compound **5a** in term of COX-1 inhibitory activity; however, its COX-2 suppression criteria was increased by factor of two (Table 1). The calculated binding mode represented in Figure 3 sheds some lights on the enhanced COX-2 affinity of the methoxy-containing derivative **5c**. The methoxy group was calculated to from two potential hydrogen-bonds with the guanidine moiety of Arg513. Intriguingly, the nitro-containing analogue showed even better COX-2 inhibitory activity and selectivity as shown in Table 1. The hypothetical binding mode of compound **5e** (Figure 3b) showed three possibilities of hydrogen-bonds formation between one oxygen atom of the nitro group and the guanido moiety of Arg513, where the oxygen atom was calculated to be 2.4 and 2.7 Å away from the two terminal nitrogen atoms of Arg513. The second oxygen atom of compound **5e** nitro group was calculated to be in the vicinity of Arg513 and the carboxylate moiety of Glu524. Figure 3 details four different potential strong hydrogen-bonds between nitro group of compound **5e** and the guanido group of Arg513 and the carboxylate moiety of Glu524.

**Figure 4:**
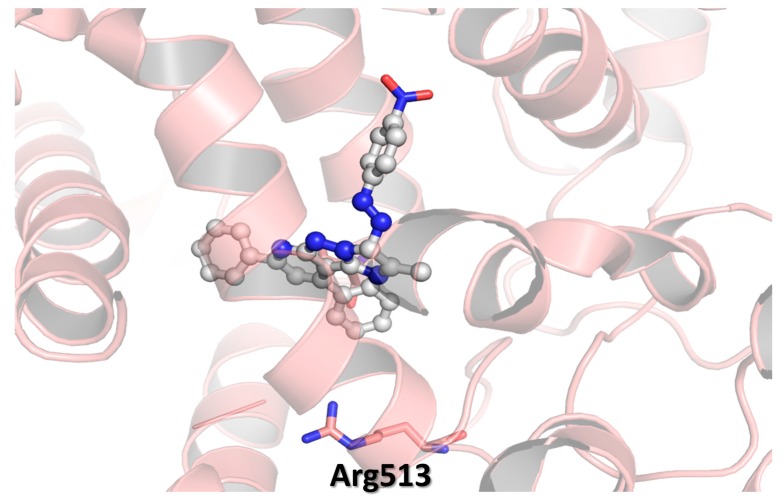
Hypothetical binding modes of compound **5i** in COX-2 protein (PDB ID:3ln1).

As mentioned earlier, COX-2 has a larger binding pocket than its isozyme-1, where three bulky amino acid residues: Ile523, His513, and Ile434 were replaced with less-sized residues (Val523, Arg513, and Val434) [27]. Therefore, increasing the molecular volume of cyclooxygenase inhibitors is a well-established strategy to reduce the binding affinity towards the less-volume binding site of COX-1. So far, we managed to build new series of COX-2 inhibitors with remarkable COX-2 selectivity when compare with diclofenac (Table 1), but, on the other hand, the best obtained selectivity did not exceed 20 times (COX-2 selectivity of compound **5e** was 19.4). This value is still less than that of celecoxib (selectivity = 30). In our attempts to improve the selectivity, we increased the bulkiness of the imidazopyrazolopyridine nucleus by replacing the methyl group(s) at positions 2, 7, and 9 with phenyl ones. Unfortunately, this approach gave less active and selective derivatives. Docking of 7,9-diphenyl analogues and 2,7,9-triphenyl analogues revealed unexpected binding modes. Figure 4 represents the hypothetical binding mode of compound **5i**, as a representative example of this series. The diazaphenyl moiety of compound **5i** was docked far away from the Arg513 residue. Unlike compound **5e**, its 7,9-diphenyl analogue has no change to interact with Arg513. That might explain why compounds **5f**–**l** have lower COX-2 inhibitory activity than their corresponding 7,9-dimethyl analogues.

#### Spectrophotometric Assay of Recombinant Human COX-2

Enzymatic activity of the purified COX-2 was measured using a chromogenic assay based on the oxidation of *N*,*N*,*N*9,*N*9-tetramethyl*p*-phenylenediamine (TMPD) during the reduction of PGG2 to PGH2 [31]. The assay mixture (180 mL) contains 100 mM sodium phosphate, pH 6.5, 1 mM hematin, 1 mg/mL gelatin, 2 to 5 mg/mL of purified COX-2, and 4 mL of the test compound in dimethyl sulfoxide (DMSO). The assay was also performed in the presence of the detergent Genapol X-100 (CalBiochem, San Diego, CA, USA) at a final concentration of 2 mM. The mixture was preincubated at room temperature (22 °C) for 15 min before initiation of the enzymatic reaction by the addition of 20 mL of a solution of 1 mM arachidonic acid and 1 mM TMPD in assay buffer (without enzyme or hematin).

For assays in the presence of Genapol, the arachidonic acid and TMPD solution was prepared in 50% aqueous ethanol. The enzyme activity was measured by estimation of the initial velocity of TMPD oxidation over the first 36 s of the reaction as followed from the increase in absorbency at 610 nm. A low rate of nonenzymatic oxidation was observed in the absence of COX-2 and was subtracted before the calculation of the percentage of inhibition.

Anti-inflammatory activity: in the same vein the anti-inflammatory activity of all prepared compounds were assayed using Rats Paw Test, where groups of adult albino rats were orally administrated with tested compounds at two different dose levels (2.5–5 mg/kg) one hour before the carrageenan challenge. Four hours after drug administration, the average weight of edema was examined, and the percentage of edema inhibition was evaluated. The well-known potassium diclofenac was used as a positive control to which the tested compounds were compared (Table 2).

The lead compound **5a** showed moderate anti-inflammatory activity in rats paw assay (Table 2). A notable correlation between COX-2 inhibitory activity (Table 1) and anti-inflammatory properties (Table 2) was observed. In general, all compounds carry hydrogen-bond acceptors para to the phenyl group attached to the diaza moiety revealed higher anti-inflammatory effect than lead compound **5a**. The most potent COX-2 inhibitor in this study (compound **5e**) revealed almost full edema protection in carrageenan-induced edema assay. The corresponding 7,9-diphenyl and 2,7,9-triphenyl analogues were less active than the corresponding 7,9-dimethylated derivatives.

Purity ≥ 95% for all synthesized compounds and this was obtained by preparative thin layer chromatography.

**Table 2:** Anti-inflammatory effect.

Compd. No.	Dose [mg/kg]	% Protection against Edema	% Inhibition of Plasma PGE2
**5a**	2.5	31.76 ± 0.66	72.19 ± 0.45
	5	40.05 ± 0.77	73.78 ± 0.43
**5b**	2.5	69.10 ± 0.85	82.65 ± 0.56
	5	71.42 ± 0.96	84.25 ± 0.45
**5c**	2.5	78.65 ± 0.87	83.59 ± 0.68
	5	88.96 ± 0.78	85.19 ± 0.79
**5d**	2.5	67.32 ± 0.89	83.13 ± 0.80
	5	70.60 ± 0.90	84.72 ± 0.97
**5e**	2.5	92.88 ± 0.97	91.73 ± 0.53
	5	97.15 ± 0.86	93.31 ± 0.46
**5f**	2.5	31.36 ± 0.55	61.28 ± 0.43
	5	33.74 ± 0.64	62.84 ± 0.56
**5g**	2.5	41.82 ± 0.75	80.82 ± 0.44
	5	44.21 ± 0.66	82.38 ± 0.21
**5h**	2.5	42.28 ± 0.57	75.49 ± 0.45
	5	54.68 ± 0.75	77.13 ± 0.89
**5i**	2.5	40.45 ± 0.63	75.96 ± 0.96
	5	42.80 ± 0.54	77.61 ± 0.89
**5j**	2.5	26.46 ± 0.45	66.45 ± 0.83
	5	28.71 ± 0.56	68.11 ± 0.83
**5k**	2.5	19.99 ± 0.67	66.93 ± 0.83
	5	22.34 ± 0.78	68.60 ± 0.98
**5l**	2.5	38.10 ± 0.69	75.01 ± 0.65
	5	40.29 ± 0.77	76.65 ± 0.34
**Diclofenac sodium**	2.5	69.59 ± 0.44	55.49 ± 0.80
	5	72.19 ± 0.54	70.59 ± 0.80
**Valdicoxib**	2.5	78.89 ± 0.33	60.09 ± 0.86
	5	87.19 ± 0.23	81.11 ± 0.21

Values were calculated from the mean values of data from three separate experiments; All results are significant different from control values at *p* ≤ 0.005; All results are significant different from reference standard values at *p* ≤ 0.005.

## 3. Experimental Protocols

### 3.1. Chemistry

#### 3.1.1. General

Melting points were measured on Electrothermal IA 9000 series digital melting point apparatus (Weiss-Gallenkamp, London, UK). The IR spectra were recorded in potassium bromide discs on a Pye Unicam SP 3300 and Shimadzu FT IR 8101 PC infrared spectrophotometer (Mattson Instruments Inc., Madison, WI, USA). ^1^H-NMR and ^13^C-NMR spectra were recorded in deuterated dimethyl sulfoxide (DMSO-*d*_6_) using a Varian Gemini 300 NMR spectrometer (300 MHz for ^1^H-NMR and 75 MHz for ^13^C-NMR, Varian, Palo Alto, CA, USA). Mass spectra were recorded on a Shimadzu GCMS-QP1000 EX mass spectrometer (Shimadzu, Tokyo, Japan) at 70 eV. Elemental analysis was carried out at the Microanalytical Centre of Cairo University, Giza, Egypt. All reactions were followed by TLC (Silica gel, Merck). 3-Amino-4,6-dimethyl-1*H*-pyrazolo[3,4-*b*]pyridine **1a** [32], 3-amino-4,6-diphenyl-1*H*-pyrazolo[3,4-*b*]pyridine **1b** [33] and hydrazonoyl halides **2** [34,35] were prepared as reported in the literature.

#### 3.1.2. General Method for the Synthesis of 2,7,9-Trisubstituted-3-(aryldiazenyl)-3*H*-imidazo[1′,2′:1,5] pyrazolo[3,4-*b*]pyridine (**5a**–**l**)

A mixture of pyrazolopyridine **1a**,**b** (1 mmol) and the appropriate hydrazonoyl halides **2** (1 mmol) in dioxane (20 mL) containing TEA (0.5 mL) was refluxed for 4–8 h (monitored by TLC), allowed to cool and the solid formed was filtered off, washed with EtOH, dried and recrystallized from DMF to give **5a**–**l**.

*2,7,9-Trimethyl-3-(phenyldiazenyl)-1H-imidazo[1′,2′:1,5]pyrazolo[3,4-b]pyridine* (**5a**). Red solid, 73% yield; m.p. 233–234 °C; IR (KBr) υ 3426 (NH), 1597 (C=N) cm^−1^; ^1^H-NMR (300 MHz, DMSO-*d*_6_): δ 2.53 (s, 3H, CH_3_), 2.78 (s, 3H, CH_3_) , 2.84 (s, 3H, CH_3_), 7.18 (s, 1H, pyridine H), 7.44–8.19 (m, 5H, Ar-H), 11.16 (s, 1H, NH); ^13^C-NMR (DMSO-*d*_6_): δ 12.6 (CH_3_), 25.8 (CH_3_), 26.7 (CH_3_), 116.9, 128.6, 128.7, 128.8, 129.2, 129.8, 132.6, 132.8, 138.1, 149.6, 156.8, 162.3 (Ar-C) ppm; MS, *m*/*z* (%) 304 (M^+^, 81), 286 (97), 247 (92), 203 (97), 171 (88), 107 (100). Anal. calcd for C_17_H_16_N_6_ (304.35): C, 67.09; H, 5.30; N, 27.61; found: 66.90; H, 5.18; N, 27.46.

*3-[(4-Chlorophenyl)diazenyl]-2,7,9-trimethyl-1H-imidazo[1′,2′:1,5]pyrazolo [3,4-b]pyridine* (**5b**). Red solid, 71% yield; m.p. 251–253 °C; IR (KBr) *υ* 3423 (NH), 1591 (C=N) cm^−1^; ^1^H-NMR (300 MHz, DMSO-*d*_6_): δ 2.56 (s, 3H, CH_3_), 2.78 (s, 3H, CH_3_) , 2.88 (s, 3H, CH_3_), 7.26 (s, 1H, pyridine H), 7.70 (d, 2H, *J* = 8.4 *Hz*, Ar-H), 8.21 (d, 2H, *J* = 8.4 *Hz*, Ar-H), 11.26 (s, 1H, NH); ^13^C-NMR (DMSO-*d*_6_): δ 12.7 (CH_3_), 26.6 (CH_3_), 27.4 (CH_3_), 125.9, 127.2, 127.8, 128.2, 128.9, 129.5, 133.6, 137.1, 144.5, 149.5, 153.1, 161.9 (Ar-C) ppm; MS, *m*/*z* (%) 340 (M^+^ + 2, 11), 338 (M^+^, 31), 302 (55), 227 (54), 198 (87), 93 (100). Anal. calcd for C_17_H_15_ClN_6_ (338.79): C, 60.27; H, 4.46; N, 24.81; found: C, 60.07; H, 4.30; N, 24.70.

*3-[(4-Methoxyphenyl)diazenyl]-2,7,9-trimethyl-1H-imidazo[1′,2′:1,5]pyrazolo[3,4-b]pyridine* (**5c**). Red solid, 73% yield; m.p. 197–199 °C; IR (KBr) υ 3388 (NH), 1601 (C=N) cm^−1^; ^1^H-NMR (300 MHz, DMSO-*d*_6_): δ 2.58 (s, 3H, CH_3_), 2.72(s, 3H, CH_3_), 2.89 (s, 3H, CH_3_), 3.82 (s, 3H, OCH_3_), 7.20 (s, 1H, pyridine H), 7.61–8.21 (m, 4H, Ar-H), 11.18 (s, 1H, NH); MS, *m*/*z* (%) 334 (M^+^, 67), 318 (77), 303 (59), 214 (52), 199 (57), 76 (100). Anal. calcd for C_18_H_18_N_6_O (334.38): C, 64.66; H, 5.43; N, 25.13; found: C, 64.84; H, 5.57; N, 25.28.

*3-[(4-Bromophenyl)diazenyl]-2,7,9-trimethyl-1H-imidazo[1′,2′:1,5]pyrazolo[3,4-b]pyridine* (**5d**). Red solid, 70% yield; m.p. 212–214 °C; IR (KBr) υ 3388 (NH), 1599 (C=N) cm^−1^; ^1^H-NMR (300 MHz, DMSO-*d*_6_): δ 2.38 (s, 3H, CH_3_), 2.53 (s, 3H, CH_3_), 2.83 (s, 3H, CH_3_), 7.13 (s, 1H, pyridine H), 7.37 (d, 2H, *J* = 8.4 *Hz*, Ar-H), 8.01 (d, 2H, *J* = 8.4 *Hz*, Ar-H), 11.07 (s, 1H, NH); ^13^C-NMR (DMSO-*d*_6_): δ 16.6 (CH_3_), 26.6 (CH_3_), 27.4 (CH_3_), 126.9, 128.6, 128.7, 128.8, 129.8, 132.5, 132.8, 138.1, 149.6, 156.8, 162.3, 166.6 (Ar-C) ppm; MS, *m*/*z* (%) 384 (M^+^ + 2, 80), 382 (M^+^, 85), 345 (100), 301 (50), 227 (55), 213 (52), 76 (52). Anal. calcd for C_17_H_15_BrN_6_ (382.05): C, 53.28; H, 3.95; N, 21.93; found: C, 53.45; H, 3.70; N, 21.70.

*2,7,9-Trimethyl-3-[(4-nitrophenyl)diazenyl]-1H-imidazo[1′,2′:1,5]pyrazolo[3,4-b]pyridine* (**5e**). Red solid, 72% yield; m.p. 237–239 °C; IR (KBr) υ 3424 (NH), 1593 (C=N) cm^−1^; ^1^H-NMR (300 MHz, DMSO-*d*_6_): δ 2.54 (s, 3H, CH_3_), 2.73 (s, 3H, CH_3_), 2.88 (s, 3H, CH_3_), 7.24 (s, 1H, pyridine H), 8.41–8.48 (m, 4H, Ar-H), 11.73 (s, 1H, NH); ^13^C-NMR (DMSO-*d*_6_): δ 13.1 (CH_3_), 25.6 (CH_3_), 26.4 (CH_3_), 120.6, 123.7, 126.6, 127.4, 127.9, 128.3, 128.9, 129.4, 129.5, 131.6, 136.2, 154.9 (Ar-C) ppm; MS, *m*/*z* (%) 349 (M^+^, 63), 304 (71), 293 (100), 288 (92), 213 (46), 157(82), 108 (80). Anal. calcd for C17H15N7O2 (349.13): C, 58.45; H, 4.33; N, 28.07; found: C, 58.25; H, 4.21; N, 28.25.

*2-Methyl-7,9-diphenyl-3-(phenyldiazenyl)-1H-imidazo[1′,2′:1,5]pyrazolo[3,4-b]pyridine* (**5f**). Brown solid, 78% yield; m.p. 211–213 °C; IR (KBr) υ 3425 (NH), 1604 (C=N) cm^−1^; ^1^H-NMR (300 MHz, DMSO-*d*_6_): δ 2.35 (s, 3H, CH_3_), 7.33–8.26 (m, 16H, Ar-H and pyridine H), 11.50 (s, 1H, NH); ^13^C-NMR (DMSO-*d*_6_): δ 13.0 (CH_3_), 116.2, 117.5, 118.7, 122.0, 125.6, 126.70, 126.1, 126.8, 128.1, 128.2, 128.9, 129.6, 129.7, 130.7, 132.1, 135.1, 137.0, 140.1, 152.7, 158.3 (Ar-C) ppm; MS, *m*/*z* (%) 428 (M^+^, 100), 351 (65), 274 (45), 259 (74), 182 (69), 77 (92). Anal. calcd for C_27_H_20_N_6_ (428.17): C, 75.68; H, 4.70; N, 19.61; found: C, 75.55; H, 4.78; N, 19.56.

*3-[(4-Methoxyphenyl)diazenyl]-2-methyl-7,9-diphenyl-1H-imidazo[1′,2′:1,5]pyrazolo[3,4-b]pyridine* (**5g**). Brown solid, 78% yield; m.p. 206–208 °C; IR (KBr) υ 3412 (NH), 1607 (C=N) cm^−1^; ^1^H-NMR (300 MHz, DMSO-*d*_6_): δ 2.24 (s, 3H, CH_3_), 3.81 (s, 3H, OCH_3_), 7.13–8.27 (m, 15H, Ar-H and pyridine H), 10.95 (s, 1H, NH); MS, *m*/*z* (%) 458 (M^+^, 88), 427 (69), 412 (85), 350 (100), 273 (90) 175 (47), 77 (95). Anal. calcd for C_28_H_22_N_6_O (458.51): C, 73.35; H, 4.84; N, 18.33; found: C, 73.50; H, 4.94; N, 18.42.

*3-((4-Chlorophenyl)diazenyl)-2-methyl-7,9-diphenyl-1H-imidazo[1′,2′:1,5]pyrazolo[3,4-b]pyridine* (**5h**). Brown solid, 70% yield; m.p. 257–259 °C; IR (KBr) υ 3408 (NH), 1599 (C=N) cm^−1^; ^1^H-NMR (300 MHz, DMSO-*d*_6_): δ 2.32 (s, 3H, CH_3_), 7.19–8.05 (m, 15H, Ar-H and pyridine H), 11.67 (s, 1H, NH); ^13^C-NMR (DMSO-*d*_6_): δ 13.9 (CH_3_), 122.0, 122.4, 127.0, 127.3, 175.5, 128.4, 129.0, 130.7, 137.7, 138.7, 140.0, 142.3, 143.8, 144.5, 145.1, 146.6, 148.1, 161.3, 167.7, 168.4 (Ar-C) ppm; MS, *m*/*z* (%) 464 (M^+^ + 2, 7), 462 (M^+^, 28), 447 (64), 427 (80), 385 (75), 256 (52), 179 (92), 77 (100). Anal. calcd for C_27_H_19_ClN_6_ (462.14): C, 70.05; H, 4.14; N, 18.15; found: C, 70.15; H, 4.19; N, 18.26.

*3-[(4-Bromophenyl)diazenyl]-2-methyl-7,9-diphenyl-1H-imidazo[1′,2′:1,5]pyrazolo[3,4-b]pyridine* (**5i**). Brown solid, 73% yield; m.p. 234–236 °C; IR (KBr) υ 3418 (NH), 1606 (C=N) cm^−1^; ^1^H-NMR (300 MHz, DMSO-*d*_6_): δ 2.41 (s, 3H, CH_3_), 7.45–8.26 (m, 15H, Ar-H and pyridine H), 11.35 (s, 1H, NH); MS, *m*/*z* (%) 509 (M^+^ + 2, 15), 507(M^+^, 18), 430 (44), 428 (64), 352 (85), 275 (72), 198 (77), 77 (100). Anal. calcd for C_27_H_19_BrN_6_ (507.38): C, 63.91; H, 3.77; N, 16.56; found: C, 63.81; H, 3.71; N, 16.47.

*2-Methyl-3-[(4-nitrophenyl)diazenyl]-7,9-diphenyl-1H-imidazo[1′,2′:1,5]pyrazolo[3,4-b]pyridine* (**5j**). Brown solid, 68% yield; m.p. 249–251 °C; IR (KBr) υ 3425 (NH), 1602 (C=N) cm^−1^; ^1^H-NMR (300 MHz, DMSO-*d*_6_): δ 2.32 (s, 3H, CH_3_), 7.13 s, 1H, pyridine H), 7.15–8.10 (m, 14H, Ar-H), 11.23 (s, 1H, NH); MS, *m*/*z* (%) 473 (M^+^, 68), 427 (49), 351 (100), 274 (66), 178 (60), 77 (95). Anal. calcd for C_27_H_19_N_7_O_2_ (473.49): C, 68.49; H, 4.04; N, 20.71; found: C, 68.61; H, 4.15; N, 20.83.

*2,7,9-Triphenyl-3-(phenyldiazenyl)-1H-imidazo[1′,2′:1,5]pyrazolo[3,4-b]pyridine* (**5k**). Brown solid, 72% yield; m.p. 264–265 °C; IR (KBr) υ 3427 (NH), 1600 (C=N) cm^−1^; 1H NMR (300 MHz, DMSO-*d*_6_): δ 6.98 (s, 1H, pyridine H), 7.01–7.38 (m, 20H, Ar-H), 11.43 (s, 1H, NH); MS, *m*/*z* (%) 490 (M^+^, 71), 413 (92), 336 (68), 259 (82), 159 (60), 77 (100). Anal. calcd for C_32_H_22_N_6_ (490.56): C, 78.35; H, 4.52; N, 17.13; found: C, 78.55; H, 4.32; N, 17.30.

*7,9-Diphenyl-3-(phenyldiazenyl)-2-(thiophen-2-yl)-1H-imidazo[1′,2′:1,5]pyrazolo[3,4-b]pyridine* (**5l**). Brown solid, 74% yield; m.p. 222–224 °C; IR (KBr) υ 3394 (NH), 1597 (C=N) cm^−1^; ^1^H-NMR (300 MHz, DMSO-*d*_6_): δ 7.11–8.42 (m, 19H, Ar-H and pyridine H), 11.39 (s, 1H, NH); MS, *m*/*z* (%) 496 (M^+^, 87), 419 (77), 413 (59), 336 (85), 259 (52), 187 (70), 77 (100). Anal. calcd for C_30_H_20_N_6_S (496.58): C, 72.56; H, 4.06; N, 16.92; found: C, 72.32; H, 4.19; N, 16.79.

#### 3.1.3. Synthesis of 2-Substituted-7,9-diphenyl-3*H*-imidazo[1′,2′:1,5]pyrazolo[3,4-*b*]pyridine (**7a**,**b**)

A mixture of 3-aminopyrazolopyridine **1b** (2.86 g, 10 mmol) and haloketone **6a**,**b** (10 mmol) in absolute EtOH (30 mL) was refluxed for 4 h. The product started to separate out during the course of reaction. The crystalline solid was filtered, washed with water, dried, and recrystallized from EtOH to give pure products **7a**,**b** respectively.

*2-Methyl-7,9-diphenyl-3H-imidazo[1′,2′:1,5]pyrazolo[3,4-b]pyridine* (**7a**). Brown solid, 78% yield; m.p. 227–229 °C; IR (KBr) υ 1608 (C=N) cm^−1^; ^1^H-NMR (300 MHz, DMSO-*d*_6_): δ 2.37 (s, 3H, CH_3_), 6.55 (s, 1H, pyridine H), 7.14–8.25 (m, 11H, Ar-H), 11.71 (s, 1H, NH); ^13^C-NMR (DMSO-*d*_6_): δ 13.7 (CH_3_), 122.0, 126.0, 126.5, 126.8, 127.9, 128.1, 128.2, 128.7, 129.5, 130.7, 132.1, 135.1, 137.0, 140.0, 146.9, 155.2 (Ar-C) ppm; MS, *m*/*z* (%) 324 (M^+^, 100), 247 (44), 232 (82), 155 (64), 77 (73). Anal. calcd for C21H16N4 (324.38): C, 77.76; H, 4.97; N, 17.27; found: C, 77.52; H, 4.85; N, 17.39.

*2,7,9-Triphenyl-1H-imidazo[1′,2′:1,5]pyrazolo[3,4-b]pyridine* (**7b**). Brown solid, 78% yield; m.p. 178–180 °C; IR (KBr) υ 1604 (C=N) cm^−1^; ^1^H-NMR (300 MHz, DMSO-*d*_6_): δ 6.92 (s, 1H, pyridine H), 7.18–8.25 (m, 16H, Ar-H), 11.07 (s, 1H, NH); MS, *m*/*z* (%) 386 (M^+^, 20), 309 (34), 232 (79), 155 (34), 77 (100). Anal. calcd for C_26_H_18_N_4_ (386.45): C, 80.81; H, 4.69; N, 14.50; found: C, 80.50; H, 4.55; N, 14.32.

#### 3.1.4. Coupling of **7a**,**b** with Benzenediazonium Chloride 

To a solution of **7a**,**b** (1 mmol) in EtOH (20 mL) sodium acetate trihydrate (0.138 g, 1 mmol) was added, and the mixture was cooled to 0–5 °C in an ice bath. The resulting cold solution was added portion wise to a cold solution of benzenediazonium chloride (prepared by diazotizing aniline) (1 mmol) dissolved in hydrochloric acid (6 M, 1 mL) with a solution of sodium nitrite (0.07 g, 1 mmol) in water (2 mL). After complete addition of the diazonium salt, the reaction mixture was stirred for a further 30 min in an ice bath. The solid that separated was filtered off, washed with water, and finally recrystallized from DMF to give product proved to be identical in all respects (m.p., mixed m.p. and IR spectra) with compounds **5f** and **5k** respectively.

### 3.2. Pharmacology

#### 3.2.1. Cyclooxygenase Inhibition Activity

The colorimetric COX (ovine) Inhibitor Screening Assay utilizes the peroxidase component of cyclooxygenase. The peroxidase activity is assayed colorimetrically by monitoring the appearance of oxidized *N*,*N*,*N*,*N*-tetramethyl-*p*-phenylenediamine (TMPD) at 590 nm. The estimation of COX-1 and COX-2 enzyme inhibitor activity was done using the kit supplied by Cayman Chemical (Ann Arbor, MI, USA). The kit contained Assay buffer (10×), Heme, COX-1 (Ovine), COX-2 (Ovine), Arachidonic acid, Potassium hydroxide, Colorimetric substrate, 96 well plate.

#### 3.2.2. Anti-Inflammatory Activity: Carrageenan-Induced Edema (Rats Paw Test)

Groups of adult male albino rats (150–180 g), each of eight animals were orally dosed with tested compounds at a dose level of 2.5–5 mg/kg, as a suspension (50% *w*/*w*) in saline and 1% tween 80, one hour before the carrageenan challenge. Foot paw edema was induced by subplantar injection of 0.05 cm^3^ of a 1% suspension of carrageenan in saline into the plantar tissue of one hind paw. An equal volume of saline was injected to the other hind paw and served as control. Four hours after drug administration, the animals were decapitated, blood was collected, and the paws were rapidly excised. The average weight of edema was examined for the treated as well as for the control group, and the percentage inhibition of weight of edema was evaluated. Diclofenac potassium (5 mg/kg) was employed as standard reference to which the tested compounds were compared.

### 3.3. Statistical Analysis

Results are expressed as mean ± S.E.M. Differences between vehicle control and treatment groups were tested using one-way ANOVA, followed by multiple comparisons by the Dunnett’s test. A value of *p* ≤ 0.005 was considered statistically significant. Dose-response curves for percent inhibition were fitted by a four-parameter logistic function using a nonlinear least-squares regression.

### 3.4. Molecular Modeling 

The structures of newly synthesized compounds were built with Sybyl-X software and minimized to 0.01 kcal/mol by the Powell method, using Gasteiger-Hückel charges and the Tripos force field [36]. The minimized molecules underwent 10 rounds of simulated annealing to search for the optimized conformation. During the simulation process, the starting conformation in each round was heated to 700 K within 1000 fs and then cooled to 200 K in the same period. Conformations were recorded at each temperature level (700 K and 200 K). The conformers located at the starting point at the each round of simulation were selected for the further energy refinement using the same parameter set as the ones in molecular construction. The minimized conformer with the lowest energy was selected as the optimized conformation molecular docking step. The proteins coordinates have been downloaded from Protein Data Bank website (PDB IDs:3ln1). The water molecules, and all other substructures including celecoxib were removed. The hydrogen atoms were added and the energy of the protein was minimized using the Amber force field with Amber charges. The energy-optimized lignads were docked into the binding site in the protein using GOLD. The parameters were set as the default values for GOLD. The maximum distance between hydrogen bond donors and acceptors for hydrogen bonding was set to 3.5 Å. After docking, the top three poses conformations of each lignad were merged into the ligands-free protein. The new ligand–protein complexes were subsequently subjected to energy minimization using the Amber force field with Amber charges. The energy minimization, in all cases, was performed using the Powell method with a 0.05 kcal/(mol Å) energy gradient convergence criterion and a distance dependent dielectric function.

## 4. Conclusions

The field of pain-control and anti-inflammation research is still suffering from several drawbacks arising from rapidly developed undesirable side effects. The clinical applications of non-steroidal anti-inflammatory drugs (NSAIDs) are hampered by suppression of COX-1 that mediates cytoprotection of gastric mucosa. Nevertheless, COX-2 is undetectable in healthy tissues and induced by inflammatory stimuli at site of inflammation. Therefore, the therapeutic benefits increase by selectively inhibiting COX-2 only. In the present study, we explored imidazopyrazolopyridine scaffold as a new potent and selective COX-2 inhibitor. Calculating the hypothetical binding mode of the lead compound **5a**, obtained by virtual screening, within COX-2 active site showed that the phenyl group connected with diaza moiety is docked in the vicinity of Arg513. Since targeting such residue is a key step in designing selective COX-2 inhibitors, several analogues with hydrogen-bond acceptors *para* to the phenyl ring were designed and built. Interestingly, all of them revealed better COX-2 inhibitory activity and selectivity as well. Compound **5e** that carries a *para* nitrophenyl group was the best COX-2 inhibitor in our series. It is 20 times more selective towards COX-2 and provided almost full edema protection in carrageenan-induced edema assay. Our attempts to improve the selectivity via increasing bulkiness around imidazopyrazolopyridine nucleus was achieved by less active compounds.

## Abbreviations

COX, cyclooxygenase; COX-1, cyclooxygenase-1; COX-2, cyclooxygenase-2; NSAIDs, non-steroidal anti-inflammatory drugs; PGs, prostaglandins.
